# Retraction Note: Glimepiride mitigates tauopathy and neuroinflammation in P301S transgenic mice: role of AKT/GSK3β signaling

**DOI:** 10.1007/s10787-025-01819-6

**Published:** 2025-07-01

**Authors:** Mennatallah O. Zaki, S. El-Desouky, Doaa A. Elsherbiny, Mohamed Salama, Samar S. Azab

**Affiliations:** 1 Department of Pharmacology and Toxicology, Faculty of Pharmacy, Horus University, New Damietta, Egypt; 2 Medical Experimental Research Center (MERC), Faculty of Medicine, Mansoura University, Mansoura, Egypt; 3 Department of Pharmacology and Toxicology, Faculty of Pharmacy, Ain Shams University, Cairo, 11566 Egypt; 4 Institute of Global Health and Human Ecology, The American University in Cairo, Cairo, Egypt

**Retraction Note: Inflammopharmacology (2022) 30:1871–1890** 10.1007/s10787-022-01023-w

The Editor-in-Chief has retracted this article. After publication, concerns were raised regarding highly similar images in Fig. 4, specifically 2nd image in the 2nd lane (Hippocampus CA1 region) in the group (WT 4 mg/kg GPD) and with 4th image in the 3rd lane (Hippocampus CA2 region) in group (P301S + 1 mg/kg GPD). An investigation by the Publisher noted irregularities in western blots for Fig. 7 provided in Supplementary Fig. 1. The authors have provided raw data but that did not address concerns fully.

The Editor-in-Chief therefore no longer has confidence in the presented data.

Mohamed Salama agrees to this retraction. Mennatallah O. Zaki does not agree to this retraction. None of the remaining authors has responded to any correspondence to the Editor regarding this retraction.

